# Virulence phenotypes result from interactions between pathogen ploidy and genetic background

**DOI:** 10.1002/ece3.6619

**Published:** 2020-08-07

**Authors:** Dorian J. Feistel, Rema Elmostafa, Meleah A. Hickman

**Affiliations:** ^1^ Department of Biology Emory University Atlanta GA USA

**Keywords:** fungi, pathogen, ploidy, virulence

## Abstract

Studying fungal virulence is often challenging and frequently depends on many contexts, including host immune status and pathogen genetic background. However, the role of ploidy has often been overlooked when studying virulence in eukaryotic pathogens. Since fungal pathogens, including the human opportunistic pathogen *Candida albicans*, can display extensive ploidy variation, assessing how ploidy impacts virulence has important clinical relevance. As an opportunistic pathogen, *C. albicans* causes nonlethal, superficial infections in healthy individuals, but life‐threatening bloodstream infections in individuals with compromised immune function. Here, we determined how both ploidy and genetic background of *C. albicans* impacts virulence phenotypes in healthy and immunocompromised nematode hosts by characterizing virulence phenotypes in four near‐isogenic diploid and tetraploid pairs of strains, which included both laboratory and clinical genetic backgrounds. We found that *C. albicans* infections decreased host survival and negatively impacted host reproduction, and we leveraged these two measures to survey both lethal and nonlethal virulence phenotypes across the multiple *C. albicans* strains. In this study, we found that regardless of pathogen ploidy or genetic background, immunocompromised hosts were susceptible to fungal infection compared to healthy hosts. Furthermore, for each host context, we found a significant interaction between *C. albicans* genetic background and ploidy on virulence phenotypes, but no global differences between diploid and tetraploid pathogens were observed.

## Graphical abstract

Virulence phenotypes depend on the genotype of both the host and the pathogen, but ploidy is an important, but understudied component of an organism's genotype. Therefore, we measured lethal and nonlethal virulence phenotypes of diploid and tetraploid pathogen, the yeast *Candida albicans*, in healthy and immunocompromised nematode hosts. We found a significant interaction between ploidy and pathogen genetic background, regardless of host immune status.

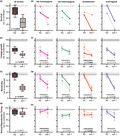

## 1 INTRODUCTION

Virulence is measured by the reduction of host fitness resulting from a host‐pathogen interaction (Cressler, McLEOD, Rozins, van den Hoogen, & Day, 2016; Lipsitch, Siller, & Nowak, 1996; Read, 1994). Therefore, virulence is not solely the property of the pathogen, but rather the product of the interaction between a host and its pathogen (Casadevall & Pirofski, 1999; Jabra‐Rizk et al., 2016). While many biotic and abiotic factors contribute to virulence (Katsir, Schilmiller, Staswick, He, & Howe, 2008; Mauch‐Mani & Mauch, 2005), the genotype‐by‐genotype interaction between hosts and pathogens is a primary determinant of whether a host gets infected and the resulting level of virulence (Lambrechts, Scott, & Gubler, 2010; Schulte, Makus, Hasert, Michiels, & Schulenburg, 2010). One important, yet understudied, element of an organism's genotype is its ploidy level. Theoretical studies demonstrate that host‐pathogens co‐evolution should drive hosts toward diploidy and pathogens toward haploidy (Nuismer & Otto, 2004). However, polyploidy may have an important role in host‐pathogen dynamics. For example, polyploidy of some host species is associated with elevated immune response (King, Seppälä, & Neiman, 2012; Osnas & Lively, 2006). Furthermore, polyploidy and aneuploidy are well‐documented in fungal pathogens that infect plant and/or animal hosts (Lin, Nielsen, Patel, & Heitman, 2008; Morrow & Fraser, 2013; Zhu, Sherlock, & Petrov, 2016). However, there have only been a limited number of studies that investigate whether pathogen ploidy impacts virulence phenotypes and the few that have, often result in contradictory findings (Hubbard, Poulter, Sullivan, & Shepherd, 1985; Ibrahim et al., 2005; Lin et al., 2008; Purnell & Martin, 1973; Suzuki, Kanbe, Kuroiwa, & Tanaka, 1986).

One potential source of these contradictory results regarding ploidy is the genetic background or allelic composition of the pathogen. Allelic composition refers to not just the specific alleles present in a genome, but the amount of heterozygosity throughout the genome. Pathogen virulence depends on the pathogen's specific allelic combination (Manning et al., 2008), and phenotypic analysis of diverse clinical isolates within pathogenic species clearly demonstrates that pathogen genetic background contributes to its virulence (Ford et al., 2015; Hirakawa et al., 2015). Ploidy intrinsically impacts allelic composition—haploids contain a single set of gene alleles, whereas diploids and polyploids can either be homozygous or heterozygous for any given locus, and dominance can mask recessive alleles. Allelic composition in polyploids is further complicated by multiple allelic ratios, in which one to four alleles may be present, depending on the mechanism and age of the polyploidization event. Thus, a major challenge in determining the specific role ploidy has on pathogen virulence is disentangling it from allelic composition.

The opportunistic fungal pathogen *Candida albicans*, while typically a highly heterozygous diploid (Abbey, Hickman, Gresham, & Berman, 2011; Jones et al., 2004; Muzzey, Schwartz, Weissman, & Sherlock, 2013), shows tremendous ploidy variation, ranging from haploid to polyploid (Gerstein, Lim, Berman, & Hickman, 2017; Hickman, Paulson, Dudley, & Berman, 2015; Hickman et al., 2013; Selmecki, Forche, & Berman, 2010) and is highly tolerant of aneuploidy (Arbour et al., 2009; Hickman et al., 2015; Lenardon & Nantel, 2012; Selmecki, Bergmann, & Berman, 2005; Selmecki, Dulmage, Cowen, Anderson, & Berman, 2009; Selmecki, Forche, & Berman, 2006). *Candida albicans* is a commensal in the human gastrointestinal microbiota and various other niches (Underhill & Iliev, 2014). Despite its commensal existence, *C. albicans* causes a range of infection, including superficial mucosal infections to life‐threatening systemic infections (Brown et al., 2012). The severity of fungal infection is closely linked to the immune status of the host, with superficial mucosal infections occurring in healthy individuals and serious bloodstream infections occurring almost exclusively in immunocompromised hosts, the latter of which has a mortality of up to 50% (Brown et al., 2012). Extensive allelic and ploidy variation, including loss‐of‐heterozygosity, aneuploidy, and polyploidy, are well‐documented in laboratory and clinical *C. albicans* isolates (Abbey et al., 2014; Abbey et al., 2011; Ford et al., 2015; Hirakawa et al., 2015; Jones et al., 2004; Muzzey et al., 2013; Ropars et al., 2018). However, these large‐scale genomic perturbations are often identified and studied in the context of antifungal drug resistance (Ford et al., 2015; Selmecki et al., 2005, 2006), and only limited studies have specifically investigated how *C. albicans* ploidy impacts virulence phenotypes (Hubbard et al., 1985; Ibrahim et al., 2005; Suzuki et al., 1986).

In this study, we sought to identify how *C. albicans* ploidy impacts its virulence by using four diploid‐tetraploid pairs of strains, with each pair representing a distinct genetic background. We assessed virulence by monitoring four measures of host fitness using healthy and immunocompromised *Caenorhabditis elegans* hosts (Feistel, 2019). While we find almost no overall relationship between ploidy and virulence, there are detectable differences among *C. albicans* genetic backgrounds and clear interactions between *C. albicans* genetic background and its ploidy state on virulence phenotypes. We also observe these interactions in immunocompromised hosts; however, in some cases, the ploidy‐specific pattern and/or the degree of virulence severity is different between healthy and immunocompromised hosts. Taken together, these results emphasize the importance of genotypic interactions on virulence phenotypes, in which genotype includes ploidy state.

## 2 MATERIAL AND METHODS

### 2.1 Strains and media

For this study, the *C. albicans* strains used are described in Table S1. *Caenorhabditis elegans* N2 Bristol (WT) and *sek‐1* mutant (Kim, 2002) were used to test host survival, fecundity and population growth in healthy and immunocompromised hosts, respectively. *Caenorhabditis elegans* populations were maintained at 20°C on lite nematode growth medium (NGM, US Biological) with the nonpathogenic *Escherichia coli* strain OP50 as a food source (Brenner, 1974) and were transferred to a newly seeded *E. coli* plate every 3–4 days. For survival, fecundity and lineage growth assays, NGM was supplemented with 0.08 g/L uridine, 0.08 g/L histidine, and 0.04 g/L adenine to facilitate growth of auxotrophic *C. albicans* strains and 0.2 g/L streptomycin sulfate to inhibit *E. coli* overgrowth so *C. albicans* strains could proliferate.

### 2.2 Survival and fecundity assays

Seeding NGM plates and *C. elegans* egg preparation for survival and fecundity assays were performed as previously described (Feistel, 2019). To establish *C. albicans* infection in nematode hosts, yeast must be ingested in the presence of *E. coli*, the standard food for laboratory *C. elegans* (Feistel, 2019; Issi, Rioux, & Rao, 2017; Jain, Pastor, Gonzalez, Lorenz, & Rao, 2013; Jain, Yun, Politz, & Rao, 2009). Briefly, *C. albicans* strains and *E. coli* OP50 were inoculated in 3 ml of YPD or 5 ml of LB, respectively, and incubated at 30°C for 1–2 days. *C. albicans* cultures were diluted to a final volume of 3.0 OD_600_ per ml, and *E. coli* was pelleted and washed twice with 1 ml of ddH_2_O. The washed pellet was centrifuged for 60 s at maximum and any excess liquid removed. The pellet was weighed and suspended with ddH_2_O to a final volume of 200 mg/ml. For uninfected treatments, 6.25 μl *E. coli* was mixed with 43.75 μl ddH_2_O. For *C. albicans* treatments, 1.25 μl *C. albicans* was mixed with 6.25 μl *E. coli* and brought to a final volume of 50 μl with ddH_2_O. All 50 μl was spotted onto the center of a 35mm supplemented‐NGM Lite agar plate and incubated at room temperature overnight before addition of nematode eggs or transferred nematode.

To synchronize host populations, *C. elegans* populations (~100 nematodes and any laid eggs) were washed off NGM plates with M9 buffer and transferred to a 15‐ml conical tube and pelleted by centrifugation for 2 min at 279 RCF. The pellet was resuspended in a 5.25% bleach solution to dissolve adult hosts and select for eggs, transferred to a microcentrifuge tube and inverted for 90–120 s and subsequently centrifuged for 30 s at 440 RCF. The pellet was washed thrice with 1 ml M9 buffer and resuspended in 500 μl M9 buffer. ~100 eggs were added to uninfected or *C. albicans* treatment plates. 48 hr later, a single L4 nematode (×10 per treatment) was randomly selected and transferred to an uninfected or *C. albicans* treatment plate and incubated at 20°C. Each founder nematode was transferred to new treatment plates every 24 hr for seven consecutive days. For survival analysis, we documented whether the founder was alive, dead, or censored (i.e., crawled off the plate or were otherwise unaccounted). For fecundity analysis, any eggs laid for each 24‐hr interval remained undisturbed on the plate and incubated at 20°C for an additional 24 hr and the number of viable progeny produced per day was scored. Total brood size is the sum of viable progeny produced over seven days. Delayed reproduction is calculated by the number of progeny produced on Day 4 or later divided by the total progeny produced for each founder nematode. All experiments were performed in triplicate or more.

### 2.3 Lineage growth assays

Lineage growth assays were performed as previously described (Feistel, 2019). In brief, a single L4 founder nematode (×6 founders per treatment) was randomly selected from a synchronized population and transferred to a 100 mm treatment plate that contained a 300 μl seed of *C. albicans* and/or *E. coli* and incubated at 20°C for five days in which the founder produces F1 and F2 progeny. The entire population derived from the single founder were washed with M9 buffer and transferred to 15‐ml conical tubes and brought to a final volume of 10 ml. Tubes were placed at 4°C for 1 hr to allow the nematodes to settle at the bottom. 20 μl samples were taken from each population and counted six independent times to determine the final population size for each founder nematode. All experiments were performed at least in duplicate.

### 2.4 Statistical analyses

All statistical analyses were performed with GraphPad Prism. Survival curves were tested for significant differences using log‐rank (Mantel–Cox) tests. Lineage growth, total brood size, and delayed reproduction data sets were tested for normality using the D'Agostino & Pearson omnibus normality test. For comparisons across genetic backgrounds, Kruskal–Wallis and Dunn's multiple comparison tests were performed. For pairwise comparisons between ploidy or host context, Mann–Whitney tests were performed. Two‐way ANOVAs were performed to test for interactions between *C. albicans* genetic background and ploidy or between *C. albicans* ploidy and host context.

## 3 RESULTS

### 3.1 Interactions between pathogen ploidy and genetic background impact virulence phenotypes in healthy hosts

**FIGURE 1:**
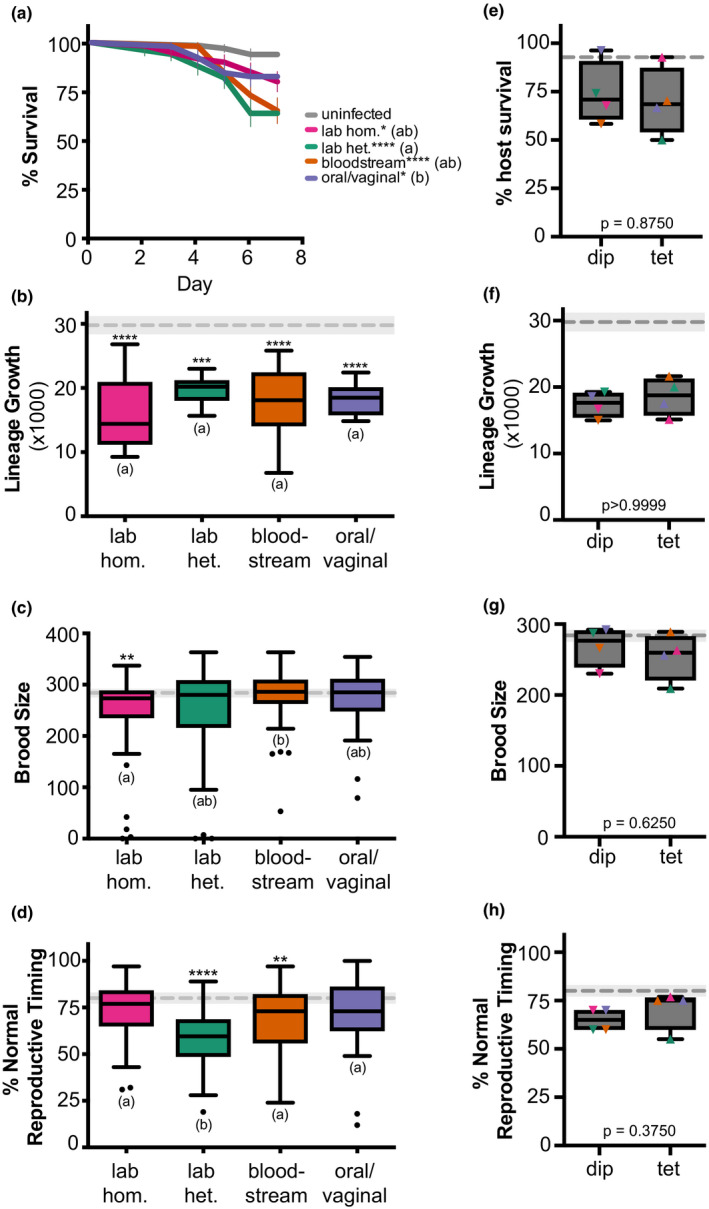
*Candida albicans* genetic background differentially impacts healthy host fitness. (a) Survival curves for healthy, wild‐type nematode host populations that were either uninfected (exposed only to an *Escherichia coli* food source, gray), or infected with different *C. albicans* strains (indicated in legend). Error bars indicated ± *SE*. The number of hosts analyzed (*n*) for each treatment is indicated in Table S1. Statistical significance was tested using pairwise comparisons of survival curves with the Log‐rank (Mantel–Cox) test. Asterisks denote statistical significance compared to the uninfected control (* indicates *p* < .05; **** indicates *p* < .0001). *Candida albicans* treatments that share letters are not significantly different, whereas treatments with differing letters are statistically different. (b) Box and whiskers plot of host lineage growth, represented by the total population size of F1 and F2 progeny from a single founder host, produced within 7 days. Boxes indicated the 25th–75th quartiles with the mean indicated as a line. Error bars are the normalized data range and circles indicate outliers. The mean and 95% CI of the uninfected control treatment is indicated by the gray dashed line and shaded gray box, respectively. Statistical significance was tested using one‐way ANOVA. Asterisks denote statistical differences compared to the uninfected control (* indicates *p* < .05; *** indicates *p* < .001). *Candida albicans* treatments that share letters are not significantly different, whereas treatments with differing letters are statistically different, post hoc Dunn's multiple comparison test. (c)Total brood size and (d) percent of host progeny produced during days 1–3 of adulthood (normal reproductive timing) of hosts infected with *C. albicans*. Data and statistical analyses are the same as (b). (e) Percent host survival on Day 7 for diploid (dip) and tetraploid (tet) *C. albicans* strains (colored symbols indicate specific *C. albicans* genetic background). Statistical significance was tested using Wilcoxon matched‐pairs signed rank test and *p*‐values are indicated. (f) Lineage growth, (g) brood size, and (h) reproductive timing of hosts infected with *C. albicans* diploid and tetraploid strains. Data and statistical analysis are the same as for (e)

First, we wanted to investigate whether *C. albicans* genetic background differentially impacted host fitness. To do this, we infected *C. elegans* hosts with four genetically distinct strain backgrounds of *C. albicans*. Two of these genetic backgrounds are laboratory strains: a “laboratory heterozygous,” which consists of the SC5314 reference strain and a “laboratory homozygous,” a derivative of SC5314 in which the genome is completely homozygous. The other two genetic backgrounds are clinical strains: a “bloodstream,” isolated from a candidemia infection of a male immunosuppressed patient and an “oral/vaginal,” a pair of clinical strains isolated from a single immune competent female patient with vulvovaginal candidiasis. For each *C. albicans* genetic background, we assessed four measures of host fitness: host survival (Figure 1a), host lineage growth (Figure 1b), host brood size (Figure 1c), and host reproductive timing (Figure 1d). For survival and lineage growth, all *C. albicans* genetic backgrounds were virulent, with significant differences observed between uninfected and infected treatments, and the only statistical difference between *C. albicans* genetic backgrounds was between the laboratory heterozygous and oral/vaginal strains for host survival. For brood size, only the laboratory homozygous genetic background was virulent and resulted in significantly smaller host brood sizes compared to the two clinical *C. albicans* backgrounds. The impact on host reproductive timing also depended on pathogen genetic background: The laboratory heterozygous and the clinical bloodstream strains were virulent (i.e., reduced amount of normal reproductive timing) whereas the other two *C. albicans* genetic backgrounds were not. The laboratory heterozygous strain impacted host reproductive timing significantly more than the three other pathogen genetic backgrounds. Together, these results suggest that while pathogen genetic background may not obviously contribute to host survival phenotypes, it may be important for other measures of fungal infection that are less lethal.

One factor that may dampen any differences in virulence among *C. albicans* genetic backgrounds is ploidy. For each genetic background, we used a related diploid strain and a tetraploid strain (Table S1). The tetraploids for both the “laboratory” strains were produced via mating in the laboratory. The “bloodstream” pair consisted of a diploid strain isolated early in the infection and its corresponding tetraploid strain was isolated mid‐to‐late infection following antifungal treatment. The “oral/vaginal” pair consisted of a diploid strain isolated from the oral cavity, and its corresponding tetraploid was isolated from the urogenital tract following antifungal treatment for vulvovaginal candidiasis. There have been limited and conflicted reports on the role of tetraploid in fungal virulence (Hubbard et al., 1985; Ibrahim et al., 2005). To assess how pathogen ploidy impacts virulence, we compared all the diploid strains to the tetraploid strains for host survival (Figure 1e), lineage growth (Figure 1f), host brood size (Figure 1g), and host reproductive timing (Figure 1h) in wild‐type hosts. We did not observe a significant difference between these two ploidy states for any of the host fitness measures tested, suggesting that ploidy may not impact virulence phenotypes. However, when we account for *C. albicans* strain background, differences between pathogen ploidy emerge but depend on strain genetic background and thus, we detect a significant interaction between genetic background and ploidy for host lineage growth (“interaction” *p* = .0354, two‐way ANOVA; Figure S1b), brood size (“interaction” *p* < .0001, two‐way ANOVA; Figure S1b), and reproductive timing (“interaction” *p* = .0283, two‐way ANOVA; Figure S1b). While we cannot directly test for an interaction using host survival curves, we do detect differences between diploids and tetraploids for each *C. albicans* genetic background (Figure S1 and Table S2). Taken together, these results suggest that there is no global pattern in ploidy state and virulence, but ploidy in combination with genetic background does significantly contribute to *C. albicans* virulence phenotypes.

**FIGURE 2:**
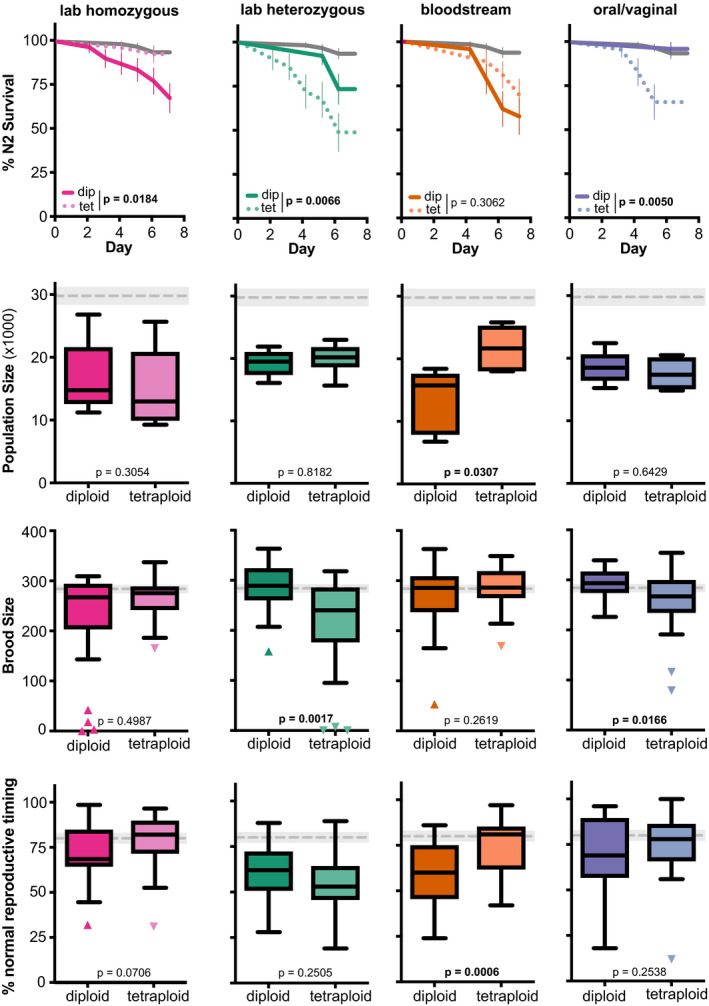
Ploidy‐specific differences across *Candida albicans* genetic backgrounds in healthy hosts. (a) Survival curves for healthy, wild‐type nematode host populations that were either uninfected (exposed only to an *Escherichia coli* food source, gray), or infected with diploid or tetraploid *C. albicans* strains from laboratory homozygous (pink), laboratory heterozygous (green), bloodstream (orange), or oral/vaginal (blue) genetic backgrounds. Error bars indicated ± *SE*. Statistical significance was tested using pairwise comparisons of diploidy and tetraploid survival curves with the Log‐rank (Mantel–Cox) test and values are indicated with significant differences highlighted in bolded text. (b) Box and whiskers plot of host lineage growth, represented by the total population size of F1 and F2 progeny from a single founder host, produced within 7 days. Boxes indicated the 25th–75th quartiles with the mean indicated as a line. Error bars are the normalized data range and circles indicate outliers. The mean and 95% CI of the uninfected control treatment is indicated by the gray dashed line and shaded gray box, respectively. Statistical significance between diploid and tetraploid strains was tested using Mann–Whitney test, and *p*‐values are indicated with significant differences highlighted in bolded text. (c) Total brood size and (d) percent of host progeny produced during days 1–3 of adulthood (normal reproductive timing) of hosts infected with diploid and tetraploid *C. albicans*. Data and statistical analyses are the same as (b)

When we look at the specific diploid‐tetraploid pairs, representing different *C. albicans* genetic backgrounds, we see significant differences in virulence between the diploid and tetraploid for at least one fitness measure, for every *C. albicans* genetic background (Figure 2). For two genetic backgrounds, the laboratory homozygous, and clinical bloodstream strains, the diploid strain was more virulent than its tetraploid counterpart. For the laboratory heterozygous and clinical oral/vaginal strains, the tetraploid strain was more virulent than its diploid counterpart, when differences between ploidy states were detected. Furthermore, these genetic background specific ploidy patterns are generally consistent across host fitness measures. For example, the laboratory heterozygous and clinical oral/vaginal tetraploids are also more virulent than their diploid counterparts for host brood size (Figure 2c), and the clinical bloodstream diploid was more virulent than its tetraploid counterpart for lineage growth and delayed host reproduction (Figure 2b,d). We performed every pairwise comparison between treatments for host survival (Table S2), lineage growth (Table S3), brood size (Table S4), and reproductive timing (Table S5) and find significant differences in virulence between different *C. albicans* genetic backgrounds and ploidy states for most host fitness measures, except host lineage growth, where very few differences between *C. albicans* strains were detected. Taken together, these data support that *C. albicans* ploidy does contribute to its virulence phenotypes, but whether it attenuates or enhances virulence depends on its genetic background.

### 3.2 Host immune status and pathogen genetic background contribute to virulence phenotypes

**FIGURE 3:**
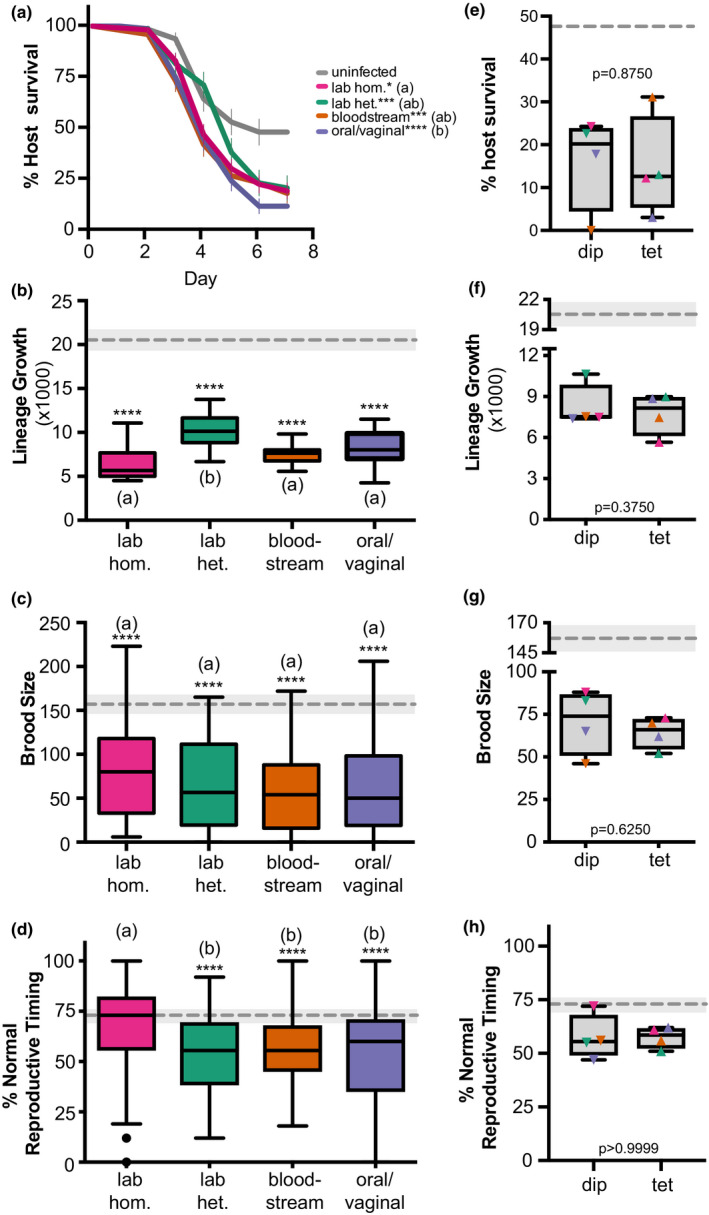
Immunocompromised hosts are highly susceptible to *Candida albicans* infection regardless of genetic background or ploidy. (a) Survival curves for immunocompromised, *sek‐1* nematode host populations that were either uninfected (exposed only to an *Escherichia coli* food source, gray), or infected with different *C. albicans* strains (indicated in legend). Error bars indicated ± *SE*. The number of hosts analyzed (*n*) for each treatment is indicated in Table S1. Statistical significance was tested using pairwise comparisons of survival curves with the Log‐rank (Mantel–Cox) test. Asterisks denote statistical significance compared to the uninfected control (* indicates *p* < .05; **** indicates *p* < .0001). *Candida albicans* treatments that share letters are not significantly different, whereas treatments with differing letters are statistically different. (b) Box and whiskers plot of host lineage growth, represented by the total population size of F1 and F2 progeny from a single founder *sek‐1* host, produced within 7 days. Boxes indicated the 25th–75th quartiles with the mean indicated as a line. Error bars are the normalized data range and circles indicate outliers. The mean and 95% CI of the uninfected control treatment is indicated by the gray dashed line and shaded gray box, respectively. Statistical significance was tested using one‐way ANOVA. Asterisks denote statistical differences compared to the uninfected control (* indicates *p* < .05; *** indicates *p* < .001). *Candida albicans* treatments that share letters are not significantly different, whereas treatments with differing letters are statistically different, post hoc Dunn's multiple comparison test. (c) Total brood size and (d) percent of host progeny produced during days 1–3 of adulthood (normal reproductive timing) of *sek‐1* hosts infected with *C. albicans*. Data and statistical analyses are the same as (b). (e) Percent *sek‐1* host survival on Day 7 for diploid (dip) and tetraploid (tet) *C. albicans* strains (colored symbols indicate specific *C. albicans* genetic background). Statistical significance was tested using Wilcoxon matched‐pairs signed rank test, and *p*‐values are indicated. (f) Lineage growth, (g) brood size, and (h) reproductive timing of *sek‐1* hosts infected with *C. albicans* diploid and tetraploid strains. Data and statistical analysis are the same as for (e)

We have previously shown that *C. albicans* and other non*albicans Candida* species cause more severe infections in an immunocompromised *C. elegans* hosts compared to healthy *C. elegans* hosts (Feistel, 2019). Given that we see pathogen ploidy patterns depend on genetic background for virulence phenotypes in wild‐type healthy hosts, we next wanted to assess if we could detect comparable patterns in immunocompromised hosts. We used a host strain with a mutation in *sek*‐1, a MAPK kinase part of the highly conserved signaling cascade required for the production of antimicrobial peptides, the main line of immune defense in *C. elegans* (Irazoqui, Urbach, & Ausubel, 2010; Kim, 2002; Kim & Ausubel, 2005). For each *C. albicans* strain, we assessed four measures of host fitness and compared *C. albicans* virulence in immunocompromised *sek‐1 C. elegans* hosts: host survival (Figure 3a), host lineage growth (Figure 3b), host brood size (Figure 3c), and host reproductive timing (Figure 3d). Nearly, all the *C. albicans* genetic backgrounds significantly reduced host fitness compared to uninfected controls, and the only exception was the laboratory homozygous strains did not significantly delay host reproduction. The laboratory homozygous background is also less virulent than the clinical oral/vaginal genetic background for host. Together, these results suggest that global differences in pathogen genetic background are revealed in hosts with compromised immune function for both lethal and nonlethal measures of host fitness.

To assess how pathogen ploidy impacts virulence in immunocompromised hosts, we compared all the diploid strains to the tetraploid strains for host survival (Figure 3e), host lineage growth (Figure 3f), host brood size (Figure 3g), and host reproductive timing (Figure 3h). Similar to healthy wild‐type hosts, we did not observe a significant difference between these two ploidy states for most of the host fitness measures. However, when we account for *C. albicans* strain background, differences between pathogen ploidy emerge but depends on strain genetic background and a significant interaction between genetic background and ploidy is detected for host lineage growth (“interaction” *p* = .0012, two‐way ANOVA; Figure S2b), brood size (“interaction” *p* = .0090, two‐way ANOVA; Figure S2c), and reproductive timing (“interaction” *p* = .0167, two‐way ANOVA; Figure S2d). While we cannot directly test for an interaction using host survival curves, we do detect differences between diploids and tetraploids for each *C. albicans* genetic background (Figure S2 and Table S2). Taken together, these results further support the observation that there is no global correlation between ploidy state and virulence, but ploidy does have an important contribution to virulence phenotypes within pathogen genetic backgrounds regardless of host immune status.

**FIGURE 4:**
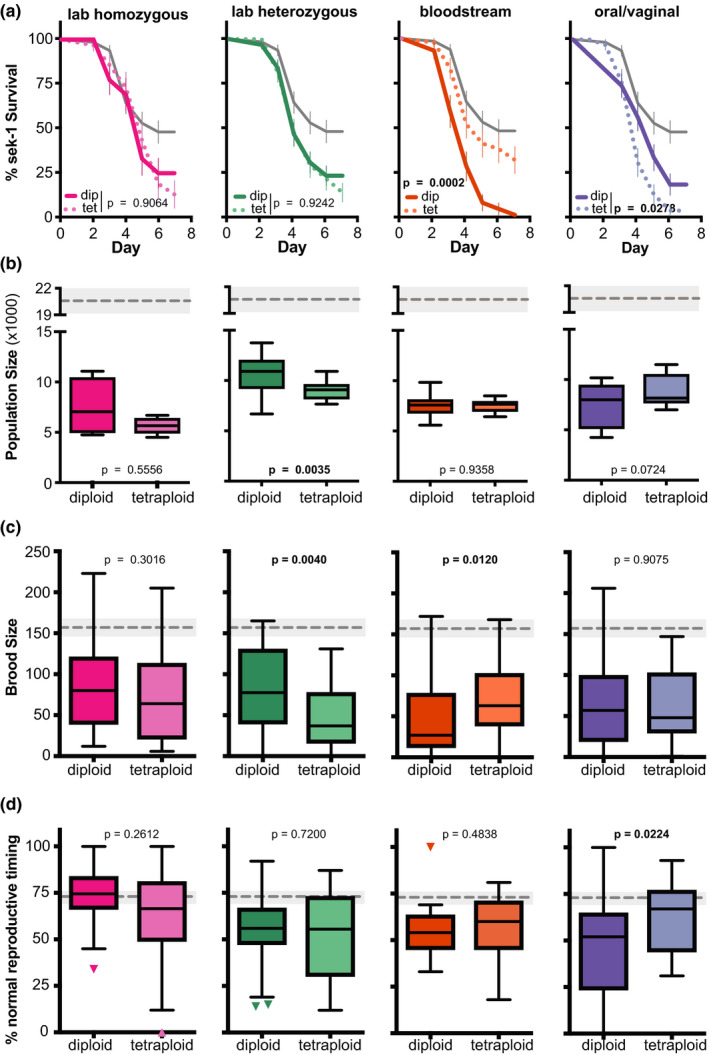
Ploidy‐specific differences across *Candida albicans* genetic backgrounds in immunocompromised hosts. (a) Survival curves for immunocompromised, *sek‐1* nematode host populations that were either uninfected (exposed only to an *Escherichia coli* food source, gray), or infected with diploid or tetraploid *C. albicans* strains from laboratory homozygous (pink), laboratory heterozygous (green), bloodstream (orange), or oral/vaginal (blue) genetic backgrounds. Error bars indicated ± *SE*. Statistical significance was tested using pairwise comparisons of diploidy and tetraploid survival curves with the Log‐rank (Mantel–Cox) test, and *p*‐values are indicated with significant differences highlighted in bolded text. (b) Box and whiskers plot of host lineage growth, represented by the total population size of F1 and F2 progeny from a single *sek‐1* founder host, produced within 7 days. Boxes indicated the 25th–75th quartiles with the mean indicated as a line. Error bars are the normalized data range and circles indicate outliers. The mean and 95% CI of the uninfected control treatment is indicated by the gray dashed line and shaded gray box, respectively. Statistical significance between diploid and tetraploid strains was tested using Mann–Whitney test, and *p*‐values are indicated with significant differences highlighted in bolded text. (c) Total brood size and (d) percent of *sek‐1* host progeny produced during days 1–3 of adulthood (normal reproductive timing) of hosts infected with diploid and tetraploid *C. albicans*. Data and statistical analyses are the same as (b)

When we look at specific diploid‐tetraploid pairs, representing different *C. albicans* genetic background, we see significant differences in virulence in immunocompromised hosts between the diploid and tetraploid state for at least one fitness measure, for all *C. albicans* genetic backgrounds except for the laboratory homozygous (Figure 4). For the laboratory heterozygous genetic background, the tetraploid counterpart was more virulent than its diploid counterpart when differences between ploidy states were detected (lineage growth and brood size), similar to the pattern observed in healthy hosts (Figure 2). Furthermore, the clinical bloodstream diploid was more virulent than its tetraploid counterpart for immunocompromised host survival and brood size (Figure 4a,c), similar to the pattern observed in healthy hosts. However, the oral/vaginal strain had significant differences between its diploid and tetraploid counterparts for host survival and reproductive timing (Figure 4a,d). The tetraploid was more virulent as measured by host survival, while the diploid was more virulent as measured by reproductive timing. We also performed every pairwise comparison between treatments for host survival (Table S2), lineage growth (Table S3), brood size (Table S4), and reproductive timing (Table S5) and find significant differences in virulence between different *C. albicans* genetic backgrounds and ploidy states for most host fitness measures, except host reproductive timing, where very few differences between *C. albicans* strains were detected. These results emphasize that while global differences between *C. albicans* genetic backgrounds or ploidy states may be difficult to identify in immunocompromised hosts (Figure 3), ploidy‐specific and genetic background differences in virulence are detectable across multiple host fitness measures.

### 3.3 Ploidy‐specific interactions between host and pathogen genotypes

**FIGURE 5:**
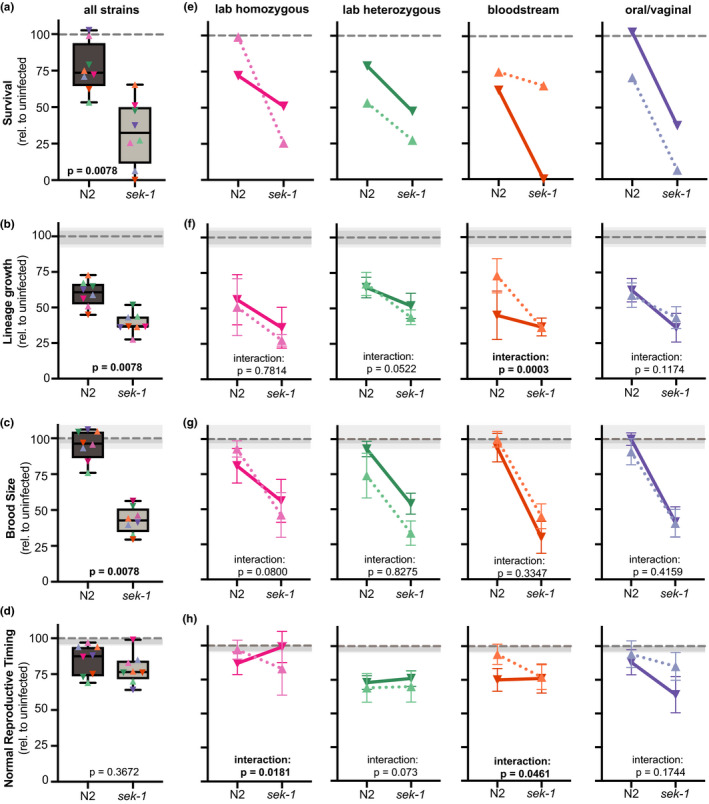
Ploidy‐specific interactions between healthy and immunocompromised hosts. (a) Relative impact on Day 7 host survival (infected/uninfected) for all *Candida albicans* strains (colored symbols indicate specific *C. albicans* genetic background) in healthy (N2) and immunocompromised (*sek‐1*) hosts. The mean and 95% CI of the uninfected control treatment are indicated by the gray dashed line and shaded box, respectively. Statistical significance between host genotypes was tested using Wilcoxon matched‐pairs signed rank test, and *p*‐values are indicated. (b) Box and whiskers plot of relative host lineage growth (c) Brood size, and (d) Reproductive timing between healthy and immunocompromised hosts. Data and statistical analysis were the same as in (a). (e) Relative impact of *C. albicans* ploidy on host survival per pathogen genetic background. Relative virulence of diploid (solid lines) and tetraploid (dotted lines) *C. albicans* laboratory homozygous (pink), laboratory heterozygous (green), bloodstream (orange), and oral/vaginal (blue) genetic background in healthy (N2) and immunocompromised (*sek‐1*) hosts. *Y*‐axis scale bar is the same as in (a). (f) Relative host lineage growth, (g) Brood size, and (h) Reproductive timing between healthy (N2) and immunocompromised (*sek‐1*) hosts across *C. albicans* genetic backgrounds. Symbols represent the mean value and error bars indicate ± one standard deviation. *Y*‐axis scale bar is the same as in (b, c, and d). Statistical significance was tested by two‐way ANOVA, and the “interaction” *p*‐values are indicated with significant differences highlighted in bolded text

Finally, we were curious if host immune status impacted the virulence relationship between each diploid‐tetraploid pair of strains. Since there are significant differences for most host fitness measures between healthy and immunocompromised hosts even when uninfected (Table S6), we normalized each *C. albicans*‐infected host fitness metric to that of the uninfected control to directly compare the severity of *C. albicans* infection between host genotypes. This analysis shows that immunocompromised hosts are significantly more susceptible to *C. albicans* infection and show larger reductions in survival, brood size, and lineage growth compared to those observed in healthy hosts, while the amount of delayed reproduction caused by *C. albicans* infection is similar (Figure 5a–d). We also detect significant interactions between *C. albicans* strain and host immune status for lineage growth (“interaction” *p* = .0004, two‐way ANOVA), brood size (“interaction” *p* = .0042, two‐way ANOVA; Figure S2c), and reproductive timing (“interaction” *p* = .0001, two‐way ANOVA).

We next examined whether the relationship between ploidy‐specific virulence differences changed depending on host immune status. To do this, we plotted the relative impact of the diploid (solid lines) and tetraploid (dotted lines) in healthy (N2) and immunocompromised (*sek‐1*) host backgrounds for all four *C. albicans* genetic backgrounds (Figure 5e‐H). In general, there was a high degree of similarity in the relationship between diploid and tetraploids across for both host genotypes, as indicated by a nonsignificant interaction term measured by two‐way ANOVA. However, there were a couple of notable exceptions, particularly in the bloodstream pair of strains. In healthy hosts, we observed the diploid displaying more severe virulence phenotypes than its tetraploid counterpart for lineage growth and reproductive timing, yet these differences are diminished in immunocompromised hosts. Thus, we detect significant interactions between host immune status and *C. albicans* ploidy for the bloodstream genetic background. Strikingly, we detect the reverse pattern for host survival with the *C. albicans* bloodstream diploid and tetraploid strains, in which no detectable differences are observed in healthy hosts, but the diploid is significantly more virulent than the tetraploid in immunocompromised hosts, yet regardless of the host context or specific fitness measure, the diploid is more virulent than its tetraploid counterpart. From these results, we conclude that interactions between host immune status, pathogen genetic background, and ploidy state determine the severity of virulence phenotypes in *C. albicans*.

## 4 DISCUSSION

Here, we sought to understand how the genetic background and ploidy of the human fungal opportunistic pathogen *C. albicans* contribute to virulence phenotypes in the nematode host *C. elegans*. Overall, we found that most of the strains we tested were virulent for at least one measure of host fitness, but cannot generalize any over‐arching patterns in how ploidy or genetic background contribute to virulence phenotypes. Rather, we have found that there is a significant interaction between *C. albicans* ploidy and its genetic background. We detect this interaction in both healthy and immunocompromised host contexts. However, immunocompromised hosts display more severe fungal infections compared to healthy hosts, regardless of *C. albicans* ploidy and/or genetic background.

*Candida albicans* was considered an “obligate” heterozygous diploid for many years (Berman & Sudbery, 2002; Noble & Johnson, 2007; Olaiya & Sogin, 1979; Riggsby, Torres‐Bauza, Wills, & Townes, 1982), and its diploid‐tetraploid parasexual cycle only discovered and characterized in the last two decades (Morrow & Fraser, 2013; Sherwood & Bennett, 2009). Much of the research regarding *C. albicans* ploidy states have focused on the mechanisms involved with the parasexual cycle and how the ploidy reduction process that tetraploids undergo promote cellular heterogeneity and genetic variation (Bennett & Johnson, 2003; Berman & Hadany, 2012; Forche et al., 2008; Gerstein et al., 2017; Hickman et al., 2015; Hirakawa, Chyou, Huang, Slan, & Bennett, 2017). There are only a small number of studies investigating if different ploidy states impact its virulence. These earlier studies investigating whether *C. albicans* ploidy impacts virulence had contradictory results—Hubbard et al demonstrated that derivative tetraploid had similar results in a murine tail vein infection model as its diploid progenitor (Hubbard et al., 1985), whereas Ibrahim et al. (2005) demonstrate that tetraploids are less virulent than diploids. Given that tetraploid *C. albicans* have been identified in clinical settings (Abbey et al., 2014) and that other fungal pathogens display extensive ploidy variation (Morrow & Fraser, 2013; Zhu et al., 2016), assessing how ploidy impacts virulence has newfound clinical relevance. We think our systematic approach of comparing paired diploid and tetraploid strain across genetic backgrounds and host contexts provides a more comprehensive analysis of ploidy and virulence. Furthermore, our results support the contradictory findings reported earlier, as we observe that sometimes certain tetraploids are less virulent than diploids, sometimes there are no differences between ploidy states, and sometimes certain tetraploids are more virulent than diploids (Tables [Link], [Link], [Link], [Link]).

Our analysis not only accounts for differences in *C. albicans* genetic background, but by utilizing *C. elegans* as an infection system we were able to assess multiple measures of host fitness beyond host survival. As an opportunistic pathogen, *C. albicans* causes a wide‐range of infections with the most severe infections occurring in immunocompromised individuals and only a fraction of these severe infections results in patient death. As such, we broadened our scope to investigate fungal virulence beyond a lethal phenotype. We previously demonstrated that *C. albicans* infection of *C. elegans* not only results in reduced survival, but also has reduced fecundity and delays in reproduction (Feistel, 2019). Here, we have leveraged this infection system to screen for differences in virulence between *C. albicans* ploidy and genetic backgrounds. While we did not find any overall pattern in virulence between diploids and tetraploids, we did identify that ploidy‐specific differences depended on genetic background (Figures 2 and 4). Importantly, these specific patterns were consistent across multiple host fitness measures and host immune status (Figure 5). Furthermore, we found that immunocompromised hosts had significantly more severe infections than immune competent hosts (Figure 5), similar to what is observed clinically.

Characterization of clinical tetraploid *C. albicans* strains has been extremely limited given how infrequently they have been isolated from infections (Abbey et al., 2014; Suzuki et al., 1986). In general, tetraploid genomes are highly unstable and often undergo parasexual ploidy reduction (Bennett & Johnson, 2003; Forche et al., 2008; Hickman et al., 2015), although rates can vary across genetic backgrounds (Gerstein et al., 2017). Ploidy reduction generates diploid derivatives that deviate from their tetraploid progenitors not only in chromosomal copy number, but allelic composition as well (Hickman et al., 2015). It is important to note that the clinical bloodstream diploid and tetraploid pair of strains are not completely isogenic and that some allelic variation exists in addition to their differences in ploidy, including altered allelic ratios on chromosomes 4, 5 and 6 (Abbey et al., 2014). It is likely that some allelic differences exist in the oral/vaginal pair of strains, although these genomes sequences have yet to be published. It is feasible that some of the differences in virulence that we observe between the diploid and tetraploids isolates in this clinical *C. albicans* backgrounds (i.e., the bloodstream diploid is more virulent than its tetraploid, whereas the oral/vaginal tetraploid is more virulent than its diploid) is due to these allelic differences. By measuring virulence phenotypes in laboratory‐derived genomes that only differ in the number of chromosome sets they contain, we can directly assess the impact ploidy has on virulence. Here, we still observe different patterns of ploidy‐specific virulence, depending on genetic background. In the laboratory heterozygous genome, we consistently observe the tetraploid as more virulent than its diploid in both healthy (Figure 2) and immunocompromised (Figure 4) host contexts. However, in the laboratory homozygous genome we frequently failed to find any significant differences between diploid and tetraploid for any of the host fitness measures, the only exception being healthy host survival, in which the tetraploid was avirulent and the diploid was virulent (Figure 2a). This result is consistent with previous findings that *C. albicans* homozygous genomes do not show significant growth differences in vitro or in vivo between ploidy states (Gerstein et al., 2017; Hickman et al., 2013).

In this work, we found that ploidy and genetic background interact to contribute to *C. albicans* virulence (Figures S1 and S2). We also observe significant interactions between *C. albicans* strains and host immune status (Figure 5). These results indicate that virulence is not simply a binary “avirulent/virulent” classification, but rather a complex trait and we need to start dissecting fungal virulence from this perspective. Recently, genome analysis of clinical isolates revealed genomic features that differ between clinical isolates and SC5314, the laboratory reference strain of *C. albicans*, and/or identify genetic determinants of antifungal drug resistance (Bensasson, 2019; Cavalieri, 2018; Ford et al., 2015; MacCallum et al., 2009; Ropars et al., 2018; Wang, Bennett, & Anderson, 2018). Only a small number have attempted to identify the genetic loci underpinning virulence, and only two genes were identified and validated, *EFG1* (Hirakawa et al., 2015; MacCallum et al., 2009) and *PHO100* (Noble & Johnson, 2007). Importantly, these approaches may overlook other factors and contexts, such as ploidy or host immune status, that contribute to virulence. While it is clear that there is variation in virulence occurs across *C. albicans* genetic backgrounds, there is still much work to do in elucidating the drivers of virulence.

## CONFLICT OF INTEREST

The author(s) declare no competing interests.

## AUTHOR CONTRIBUTION

**Dorian J Feistel:** Conceptualization (equal); Data curation (equal); Formal analysis (equal); Methodology (equal). **Rema Elmostafa:** Data curation (equal); Formal analysis (equal). **Meleah Hickman:** Conceptualization (equal); Formal analysis (equal); Funding acquisition (equal); Supervision (equal); Visualization (equal); Writing‐original draft (equal); Writing‐review & editing (equal).

## Supporting information

Figure S1

Figure S2

Table S1

Table S2

Table S3

Table S4

Table S5

Table S6

## Data Availability

All relevant data is posted at the Dryad Digital Repository (https://doi.org/10.5061/dryad.dz08kprvk).
