# Supplementary material for: Virulence phenotypes result from interactions between pathogen ploidy and genetic background
Source: Ecol Evol. 2020 Aug 7;10(17):9326–38. doi: 10.1002/ece3.6619 (PMC7487253; doi:10.1002/ece3.6619)
Supplement: Supplementary file 2 — Figure S2 [file ECE3-10-9326-s002.pdf]

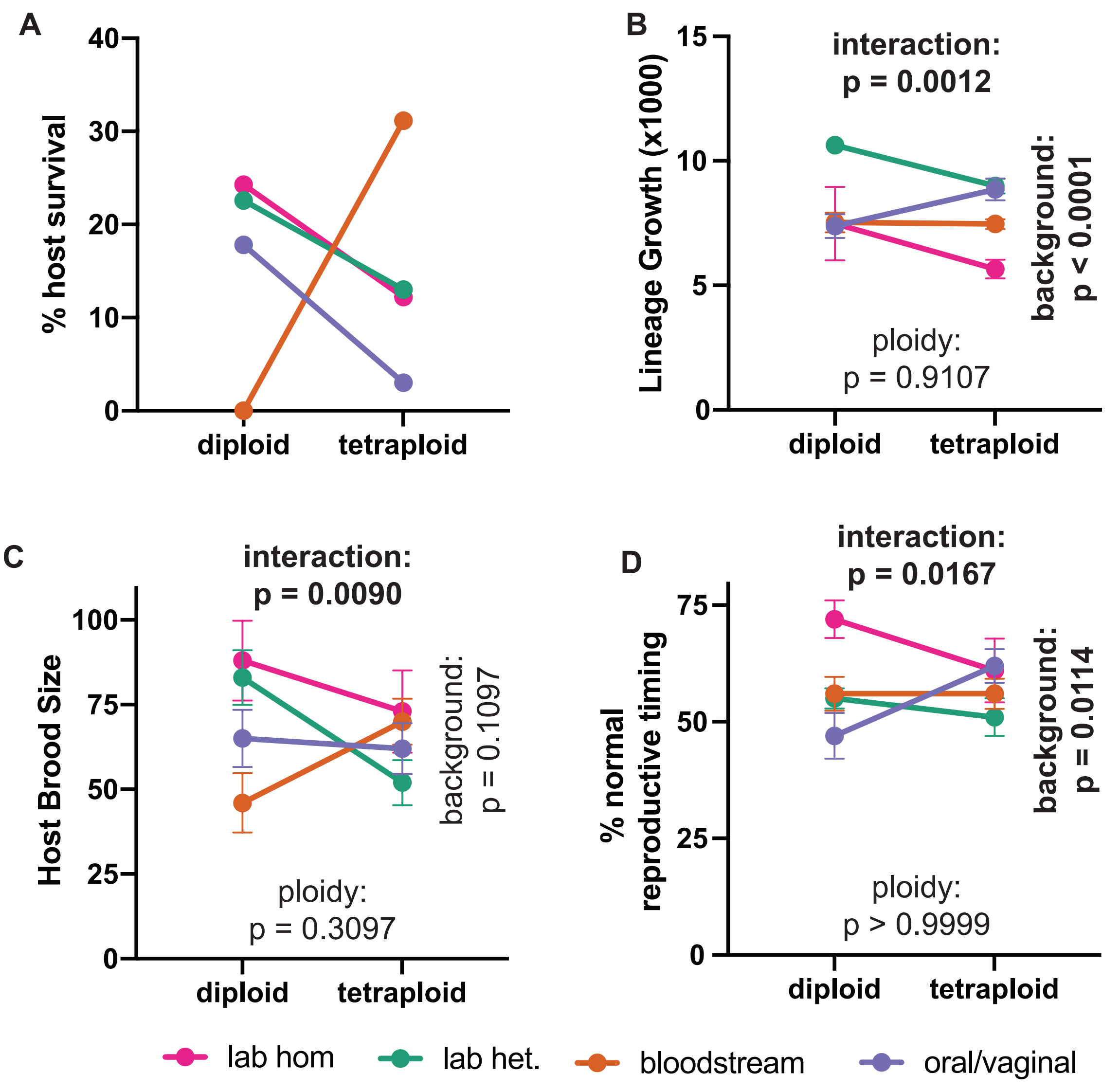

Figure S2: Interaction between *C. albicans* ploidy and genetic background on virulence phenotypes in immunocompromised hosts.
A) Relationship of Day 7 host survival between diploid or tetraploid *C. albicans* of the lab homozygous (pink), lab heterozygous (green), bloodstream (orange), and oral/vaginal (blue) genetic backgrounds in immunocompromised (*sek-1*) hosts.
B) Relationship of host lineage growth, **C)** brood size, and **D)** reproductive timing between diploid or tetraploid *C. albicans* of the lab homozygous (pink), lab heterozygous (green), bloodstream (orange), and oral/vaginal (blue) genetic backgrounds in immunocompromised (*sek-1*) hosts. Symbols represent the mean value and error bars +/-SD. Stastistical significance was tested by two-way ANOVA and p values for 'ploidy,' 'genetic background,' and their 'interaction' is indicated.
