# Supplementary material for: Virulence phenotypes result from interactions between pathogen ploidy and genetic background
Source: Ecol Evol. 2020 Aug 7;10(17):9326–38. doi: 10.1002/ece3.6619 (PMC7487253; doi:10.1002/ece3.6619)
Supplement: Supplementary file 3 — Table S1 [file ECE3-10-9326-s003.pdf]

Strain	Alias	Ploidy	Genetic Background	Source
YJB12804	lab hom.	diploid	Laboratory SC5314-derived homozygous genome (auto-diploid of YJBXXX)	Hickman et al 2013; Gerstein et al (2017)
MH306	lab hom.	tetraploid	Mating product between two SC5314-derived homozygous strains	This study
SC5314	lab het.	diploid	Laboratory reference strain	Gillum et al 1984
RBY18	lab het.	tetraploid	Mating product between two SC5314-derived heterozygous strains	Bennett and Johnson 2003
FH1	bloodstream	diploid	Clinical isolate from marrow transplant patient	Hull et al Marr et al (1997); Abbey et al (2014)
FH6	bloodstream	~tetraploid	Clinical isolate from marrow transplant patient (same patient as FH1)	Hull et al Marr et al (1997); Abbey et al (2014)
PN2	oral/vaginal	diploid	Clinical isolate recovered from oral cavity	Gerstein et al (2017)
PN1	oral/vaginal	tetraploid	Clinical isolate recovered from vaginal infection (same patient as PN2)	Gerstein et al (2017)

Table S1. Strains used in this study
