# Supplementary material for: Virulence phenotypes result from interactions between pathogen ploidy and genetic background
Source: Ecol Evol. 2020 Aug 7;10(17):9326–38. doi: 10.1002/ece3.6619 (PMC7487253; doi:10.1002/ece3.6619)
Supplement: Supplementary file 4 — Table S2 [file ECE3-10-9326-s004.pdf]

Strain 1	Strain 2	Healthy hosts p-value	Immunocompromised hosts p-value
uninfected	2C Lab hom	*** **(0.0003)**	ns (0.0679)
	4C Lab hom	ns (0.7882)	* **(0.0484)**
	2C Lab het	**** **(<0.0001)**	** **(0.0017)**
	4C lab het	**** **(<0.0001)**	*** **(0.0005)**
	2C bloodstream	**** **(<0.0001)**	**** **(<0.0001)**
	4C bloodstream	** **(0.0015)**	ns (0.0777)
	2C oral/vag	ns (0.6802)	** **(0.0029)**
	4C oral/vag	*** **(0.0002)**	**** **(<0.0001)**
2C Lab hom	4C Lab hom	* **(0.0184)**	ns (0.9065)
	2C Lab het	* **(0.0175)**	ns (0.6837)
	4C lab het	*** **(0.0002)**	ns (0.2758)
	2C bloodstream	ns (0.5167)	*** **(0.0006)**
	4C bloodstream	ns (0.7351)	ns (0.8526)
	2C oral/vag	** **(0.0060)**	ns (0.5026)
	4C oral/vag	ns (0.8687)	** **(0.0076)**
4C Lab hom	2C Lab het	**** **(<0.0001)**	ns (0.8114)
	4C lab het	**** **(<0.0001)**	ns (0.3901)
	2C bloodstream	** **(0.0038)**	*** **(0.0004)**
	4C bloodstream	* **(0.0376)**	ns (0.6867)
	2C oral/vag	ns (0.5683)	ns (0.5807)
	4C oral/vag	* **(0.0164)**	** **(0.0050)**
2C lab het	4C lab het	** **(0.0066)**	ns (0.9242)
	2C bloodstream	ns (0.0985)	**** **(<0.0001)**
	4C bloodstream	** **(0.0061)**	ns (0.4505)
	2C oral/vag	**** **(<0.0001)**	ns (0.6232)
	4C oral/vag	ns (0.0620)	** **(0.0019)**
4C lab het	2C bloodstream	** **(0.0019)**	** **(0.0043)**
	4C bloodstream	**** **(<0.0001)**	ns (0.1528)
	2C oral/vag	**** **(<0.0001)**	ns (0.7079)
	4C oral/vag	** **(0.0014)**	* **(0.0477)**
2C bloodstream	4C bloodstream	ns (0.3062)	*** **(0.0002)**
	2C oral/vag	*** **(0.0009)**	** **(0.0021)**
	4C oral/vag	ns (0.7936)	ns (0.2983)
4C bloodstream	2C oral/vag	* **(0.0114)**	ns (0.3112)
	4C oral/vag	ns (0.5600)	** **(0.0029)**
2C oral/vag	4C oral/vag	** **(0.0050)**	* **(0.0278)**

Table S2: Pairwise survival curve comparisons (log-rank test) for uninfected and
