# Supplementary material for: Virulence phenotypes result from interactions between pathogen ploidy and genetic background
Source: Ecol Evol. 2020 Aug 7;10(17):9326–38. doi: 10.1002/ece3.6619 (PMC7487253; doi:10.1002/ece3.6619)
Supplement: Supplementary file 5 — Table S3 [file ECE3-10-9326-s005.pdf]

Strain 1	Strain 2	Healthy hosts	Immunocompromised hosts
		p-value	p-value
uninfected	2C Lab hom	****** (<0.0001)**	****** (<0.0001)**
	4C Lab hom	****** (<0.0001)**	****** (<0.0001)**
	2C Lab het	****** (<0.0001)**	****** (<0.0001)**
	4C lab het	****** (<0.0001)**	****** (<0.0001)**
	2C bloodstream	****** (<0.0001)**	****** (<0.0001)**
	4C bloodstream	***** (0.0006)**	****** (<0.0001)**
	2C oral/vag	****** (<0.0001)**	****** (<0.0001)**
	4C oral/vag	***** (0.0003)**	****** (<0.0001)**
2C Lab hom	4C Lab hom	ns (0.3054)	ns (0.5556)
	2C Lab het	ns (0.2198)	*** (0.0282)**
	4C lab het	ns (0.1471)	ns (0.4605)
	2C bloodstream	ns (0.5135)	ns (0.8513)
	4C bloodstream	ns (0.0753)	ns (>0.9999)
	2C oral/vag	ns (0.2635)	ns (0.8313)
	4C oral/vag	ns (0.5395)	ns (0.4626)
4C Lab hom	2C Lab het	ns (0.2520)	****** (<0.0001)**
	4C lab het	ns (0.1120)	***** (0.0002)**
	2C bloodstream	ns (0.4222)	**** (0.0087)**
	4C bloodstream	ns (0.0709)	***** (0.0009)**
	2C oral/vag	ns (0.3002)	ns (0.1341)
	4C oral/vag	ns (0.5185)	***** (0.0002)**
2C lab het	4C lab het	ns (0.8182)	**** (0.0035)**
	2C bloodstream	*** (0.0303)**	****** (<0.0001)**
	4C bloodstream	ns (0.3290)	****** (<0.0001)**
	2C oral/vag	ns (0.6991)	****** (<0.0001)**
	4C oral/vag	ns (0.2857)	ns (0.2439)
4C lab het	2C bloodstream	*** (0.0303)**	**** (0.0069)**
	4C bloodstream	ns (0.5368)	***** (0.0004)**
	2C oral/vag	ns (0.2403)	*** (0.0396)**
	4C oral/vag	ns (0.2571)	ns (0.5698)
2C bloodstream	4C bloodstream	*** (0.0317)**	ns (0.9358)
	2C oral/vag	ns (0.0823)	ns (0.9241)
	4C oral/vag	ns (0.2857)	*** (0.0490)**
4C bloodstream	2C oral/vag	ns (0.3290)	ns (0.8240)
	4C oral/vag	ns (0.1905)	*** (0.0147)**
2C oral/vag	4C oral/vag	ns (0.6429)	ns (0.0724)

Table S3: Pairwise lineage growth comparisons (lMann Whitney test) for uninfe
