# Supplementary material for: Virulence phenotypes result from interactions between pathogen ploidy and genetic background
Source: Ecol Evol. 2020 Aug 7;10(17):9326–38. doi: 10.1002/ece3.6619 (PMC7487253; doi:10.1002/ece3.6619)
Supplement: Supplementary file 6 — Table S4 [file ECE3-10-9326-s006.pdf]

Strain 1	Strain 2	Healthy hosts	Immunocompromised hosts
		p-value	p-value
uninfected	2C Lab hom	**** (0.0023)**	****** (<0.0001)**
	4C Lab hom	*** (0.0246)**	****** (<0.0001)**
	2C Lab het	ns (0.4481)	****** (<0.0001)**
	4C lab het	***** (0.0004)**	****** (<0.0001)**
	2C bloodstream	ns (0.5219)	****** (<0.0001)**
	4C bloodstream	ns (0.5210)	****** (<0.0001)**
	2C oral/vag	ns (0.2424)	****** (<0.0001)**
	4C oral/vag	ns (0.0511)	****** (<0.0001)**
2C Lab hom	4C Lab hom	ns (0.4987)	ns (0.3016)
	2C Lab het	**** (0.0068)**	ns (0.7813)
	4C lab het	ns (0.3550)	*** (0.0120)**
	2C bloodstream	ns (0.1106)	**** (0.0038)**
	4C bloodstream	**** (0.0027)**	ns (0.2498)
	2C oral/vag	**** (0.0012)**	ns (0.1265)
	4C oral/vag	ns (0.4865)	ns (0.1646)
4C Lab hom	2C Lab het	*** (0.0257)**	ns (0.4235)
	4C lab het	ns (0.0637)	ns (0.1784)
	2C bloodstream	ns (0.3451)	*** (0.0500)**
	4C bloodstream	*** (0.0314)**	ns (0.9242)
	2C oral/vag	***** (0.0061)**	ns (0.5840)
	4C oral/vag	ns (0.9819)	ns (0.7950)
2C lab het	4C lab het	**** (0.0017)**	***** (0.0010)**
	2C bloodstream	ns (0.3270)	**** (0.0021)**
	4C bloodstream	ns (0.9897)	ns (0.2655)
	2C oral/vag	ns (0.7946)	ns (0.0817)
	4C oral/vag	ns (0.0620)	ns (0.0746)
4C lab het	2C bloodstream	*** (0.0353)**	ns (0.2879)
	4C bloodstream	**** (0.0012)**	*** (0.0364)**
	2C oral/vag	***** (0.0002)**	ns (0.3985)
	4C oral/vag	ns (0.1231)	ns (0.2828)
2C bloodstream	4C bloodstream	ns (0.2619)	*** (0.0102)**
	2C oral/vag	ns (0.1678)	ns (0.1165)
	4C oral/vag	ns (0.4100)	*** (0.0497)**
4C bloodstream	2C oral/vag	ns (0.8151)	ns (0.4683)
	4C oral/vag	*** (0.0393)**	ns (0.3723)
2C oral/vag	4C oral/vag	*** (0.0166)**	ns (0.9075)

Table S4: Pairwise brood size comparisons (lMann Whitney test) for uninfected
