# Supplementary material for: Virulence phenotypes result from interactions between pathogen ploidy and genetic background
Source: Ecol Evol. 2020 Aug 7;10(17):9326–38. doi: 10.1002/ece3.6619 (PMC7487253; doi:10.1002/ece3.6619)
Supplement: Supplementary file 7 — Table S5 [file ECE3-10-9326-s007.pdf]

Strain 1	Strain 2	Healthy hosts (N2)	Immunocompromised hosts (sek-1)
		p-value	p-value
uninfected	2C Lab hom	** (0.0062)	ns (0.8702)
	4C Lab hom	ns (0.4611)	ns (0.1466)
	2C Lab het	**** (<0.0001)	**** (<0.0001)
	4C lab het	**** (<0.0001)	**** (<0.0001)
	2C bloodstream	**** (<0.0001)	**** (<0.0001)
	4C bloodstream	ns (0.2276)	**** (<0.0001)
	2C oral/vag	* (0.0235)	**** (<0.0001)
	4C oral/vag	ns (0.2811)	** (0.0065)
2C Lab hom	4C Lab hom	ns (0.0706)	ns (0.2612)
	2C Lab het	* (0.0147)	*** (0.0002)
	4C lab het	** (0.0051)	** (0.0017)
	2C bloodstream	* (0.0309)	** (0.0021)
	4C bloodstream	ns (0.1884)	** (0.0025)
	2C oral/vag	ns (0.9492)	*** (0.0003)
	4C oral/vag	ns (0.1728)	ns (0.0671)
4C Lab hom	2C Lab het	**** (<0.0001)	ns (0.1135)
	4C lab het	**** (<0.0001)	ns (0.1660)
	2C bloodstream	**** (<0.0001)	ns (0.1362)
	4C bloodstream	ns (0.7349)	ns (0.2955)
	2C oral/vag	ns (0.1165)	ns (0.1023)
	4C oral/vag	ns (0.7460)	ns (0.8598)
2C lab het	4C lab het	ns (0.2505)	ns (0.7200)
	2C bloodstream	ns (0.9813)	ns (0.7254)
	4C bloodstream	*** (0.0002)	ns (0.6838)
	2C oral/vag	* (0.0267)	ns (0.2165)
	4C oral/vag	*** (0.0002)	ns (0.1229)
4C lab het	2C bloodstream	ns (9.3855)	ns (0.9048)
	4C bloodstream	*** (0.0005)	ns (0.5095)
	2C oral/vag	** (0.0034)	ns (0.5684)
	4C oral/vag	*** (0.0002)	ns(0.0953)
2C bloodstream	4C bloodstream	*** (0.0006)	ns (0.4838)
	2C oral/vag	* (0.0486)	ns (0.4857)
	4C oral/vag	** (0.0013)	ns (0.1824)
4C bloodstream	2C oral/vag	ns (0.3885)	ns (0.1408)
	4C oral/vag	ns (0.9500)	ns (0.2732)
2C oral/vag	4C oral/vag	ns (0.2538)	* (0.0224)

Table S5: Pairwise reproductive timing comparisons (lMann Whitney test) for ur
