# Supplementary material for: Virulence phenotypes result from interactions between pathogen ploidy and genetic background
Source: Ecol Evol. 2020 Aug 7;10(17):9326–38. doi: 10.1002/ece3.6619 (PMC7487253; doi:10.1002/ece3.6619)
Supplement: Supplementary file 8 — Table S6 [file ECE3-10-9326-s008.pdf]

		uninfected	Laboratory		Oral/Vaginal		Bloodstream	
			diploid (MH88)	tetraploid (MH130)	diploid (PN2)	tetraploid (PN1)	diploid (FH1)	tetraploid (FH6)
% Brood Size (rel. to uninfect)	Healthy (N2)	**100**±2.3 (n=36)	**100**±2.8 (n=35)	**74**±7.6 (n=22)	**100**±2.1 (n=26)	**91**±4.6 (n=24)	**94**±4.8 (n=24)	**100**±2.8 (n=27)
	Immunocompromised (sek-1)	**100**±3.9 (n=60)	**47**±4.4 (n=57)	**33**±4.3 (n=40)	**42**±5.4 (n=41)	**40**±4.8 (n=33)	**30**±5.6 (n=29)	**45**±4.4 (n=38)
	p-value	nd	****	****	****	****	****	****
% Late Reproduction (rel to total progeny)	Healthy (N2)	**23**±2.1 (n=36)	**43**±2.3 (n=35)	**45**±4.0 (n=19)	**30**±3.6 (n=26)	**25**±3.8 (n=23)	**40**±3.3 (n=24)	**25**±3.0 (n=27)
	Immunocompromised (sek-1)	**26**±2.0 (n=55)	**44**±2.5 (n=47)	**49**±4.1 (n=32)	**53**±4.8 (n=30)	**38**±3.6 (n=25)	**44**±3.7 (n=17)	**44**±3.3 (n=31)
	p-value	ns	ns	ns	****	ns	ns	***
% Lineage Expansion (rel. to uninfect)	Healthy (N2)	**100**±2.5 (n=11)	**63**±2.9 (n=6)	**66**±3.3 (n=6)	**61**±3.3 (n=6)	**57**±4.2 (n=4)	**49**±6.6 (n=4)	**71**±5.2 (n=5)
	Immunocompromised (sek-1)	**100**±3.4 (n=28)	**53**±1.5 (n=29)	**44**±1.4 (n=13)	**36**±2.3 (n=19)	**43**±2.1 (n=13)	**37**±1.9 (n=11)	**36**±1.0 (n=11)
	p-value	nd	*	****	*	****	****	*

Table S6: Pairwise comparisons (Mann Whitney test) between healthy (N2) and immunocompromised (sek-1) hosts for uninfected and all C. albicans treatments.
